# Recent Applications of Interfacial Exciplex as Ideal Host of Power-Efficient OLEDs

**DOI:** 10.3389/fchem.2019.00306

**Published:** 2019-05-07

**Authors:** Baohua Zhang, Zhiyuan Xie

**Affiliations:** ^1^Center for Advanced Analytical Science, School of Chemistry and Chemical Engineering, Guangzhou University, Guangzhou, China; ^2^State Key Laboratory of Polymer Physics and Chemistry, Changchun Institute of Applied Chemistry, Chinese Academy of Sciences, Changchun, China

**Keywords:** OLED, exciplex, thermally activated delayed fluorescence, phosphorescence, power efficiency

## Abstract

Currently, exploring the applications of intermolecular donor-acceptor exciplex couple as host of OLEDs with phosphorescence, thermally activated delayed fluorescence (TADF) or fluorescence emitter as dopant is a hot topic. Compared to other host strategies, interfacial exciplex has the advantage in various aspects, such as barrier-free charge injection, unimpeded charge transport, and the energy-saving direct exciton formation process at the “Well”-like heterojunction interface region. Most importantly, due to a very fast and efficient reverse intersystem-crossing (RISC) process, such a host is capable of regulating singlet/triplet exciton populations in itself as well as in the dopant emitters both under photoluminescent (PL) and electroluminescent (EL) driving conditions. In this mini-review, we briefly summarize and comment on recent applications of this ideal host in OLEDs (including both thermal-evaporation OLEDs and solution-processed OLEDs) with diverse emitters, e.g., fluorescence, phosphorescence, delayed fluorescence, or others. Special attention is given to illustrate the peculiar achievement of high overall EL performance with superiorities of low driving voltages, slow roll-off rate, high power efficiencies and satisfied device lifetime using this host strategy, which is then concluded by personal perspectives on the relevant next-step in this field.

Since their invention in 1987 (Tang and Vanslyke, 1987), OLEDs have received persistent attention considering their great advantage in modern displays and lighting applications (Burroughes et al., 1990; Kido et al., 1995). With the development of phosphorescent emitters (Baldo et al., 1998), efficiencies of OLEDs have been significantly improved (Wang et al., 2018). Endo et al. (2011) launched new generation OLEDs by inventing efficient thermal-activated delayed fluorescence (TADF) emitters purely from aromatic carbon materials. On the basis of a high RISC rate and a high radiative decay rate (S_1_ → *S*_0_) in TADF emitters (Uoyama et al., 2012; Higuchi et al., 2015; Noda et al., 2018), or exciplex couples (Goushi and Adachi, 2012; Goushi et al., 2012; Hung et al., 2013, 2014; Liu et al., 2015a,c), highly efficient monochromatic and white TADF OLEDs have been achieved. Other candidates such as hybridized local and charge-transfer (HLCT) excited state molecules (Li et al., 2014) and radical-based double emission molecules (Ai et al., 2018) have also been reported and well-documented, showing the analogous cost and performance merits. However, irrespective of emitter categories, e.g., phosphorescent, TADF or fluorescent emitters, it is highly pursued ideal hosts that maximize their EL performance since critical parameters of OLEDs e.g., external quantum efficiency (EQE), power efficiency (PE), roll-off rate and device lifetime, are highly determined by host choices. Among those host strategies (Tao et al., 2011; Yook and Lee, 2014; Wang et al., 2019), interfacial exciplex seems an ideal choice since all these expected characteristics are simultaneously satisfied. Based on relevant publications and our understanding, this mini-review presents a short introduction on its application status and remarks on the future research direction.

## Interfacial Exciplex as Host in Thermal-Evaporated OLEDs

**Figure 1:**
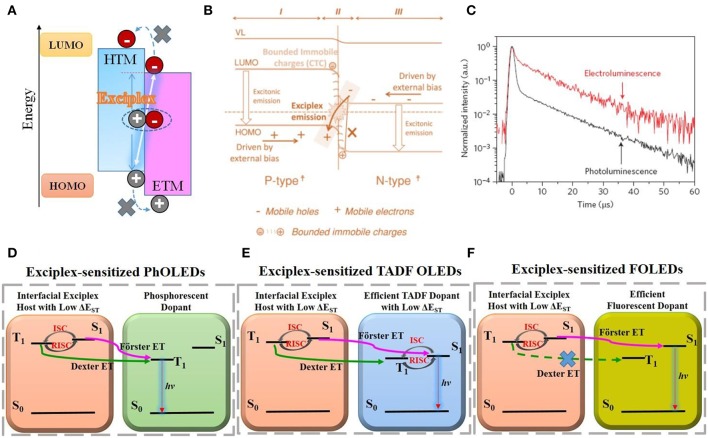
**(A)** Schematic illustration on interfacial exciplex formation. **(B)** Electronic energy-level alignments of exciplex-forming P/N heterojunction mentioned in Ng et al. (2014). Reprinted with permission from Ng et al. (2014), Copyright 2014 Wiley-VCH. **(C)** EL/PL transient of m-MTDATA:t-Bu-PBD exciplex shown in Goushi et al. (2012). Reprinted by permission from Goushi et al. (2012), Copyright 2012 Springer Nature. **(D–F)** exciplex-to-dopant ET process in exciplex-sensitized PhOLEDs, TADF OLEDs, and FOLEDs, respectively.

At a type II P/N organic/organic (O/O) heterojunction interface between an electron-donating molecule and an electron-accepting molecule, there is a high tendency to form a charge-transfer excited-state complex, also known as an exciplex (Jenekhe and Osaheni, 1994; Itano et al., 1998; Giro et al., 2000; Morteani et al., 2003). Simultaneously, HOMO and LUMO levels of hole transporting material (HTM) and electron transporting material (ETM) display a distinct gap at the heterojunction interface (Figure 1A). It is barely possible to generate exciton on either constituting molecule. By contrast, exciplex formation is energetically allowed, in which one of them locates in the excited state while another one is in the ground state being coupled. There is an experimental guideline on exciplex formation (Matsumoto et al., 2008), i.e., coexistence of huge gap, e.g., larger than 0.3 eV, for both HOMO and LUMO levels. However, it is not necessarily the case. A certain constituting material couple could be switched to exciplex or not simply by altering the substrate (Ng et al., 2014). From the electronic viewpoint, Ng et al. (2014) illustrated how the local molecular interactions and interfacial energetics at PN heterojunction play a role in exciplex formation (Figure 1B). It corresponds to P^δ−^-N^δ+^ contact at the PN heterojunction, in which the N-type material donates electrons to the LUMO level of the P-type material at the interface. Bounded immobile charges (CTC) formed thus guarantee the exciplex formation. From the classic viewpoint of semiconductor physics, exciplex is a universal concept, i.e., including organic exciplex but not limited to it, such as hybrid exciplex in a lead halide perovskite (MAPbI_3−x_Cl_x_)/quantum dot (core/shell PbS/CdS) heterojunction (Sanchez et al., 2016).

Previously, exciplex was frequently observed during fabrication of thermal-evaporated OLEDs (e-OLEDs), but unwelcomed due to its low PL quantum efficiency (PLQE) as an emitter and quenching effect in host-guest-doping devices. For instance, interfacial exciplex was discovered (or generated but not noticed by researchers) at the interface between the emissive layer (EML) and hole transporting layer (HTL), or between the EML and electron transporting layer (ETL). As the singlet and triplet levels (S_1_/T_1_) of the exciplex were less than those of emissive constituents of the EML, EL spectra and device efficiency were deteriorated via a back-energy transfer from them to the exciplex (Jenekhe and Osaheni, 1994; Gebler et al., 1997, 1998; Itano et al., 1998; Giro et al., 2000; Matsumoto et al., 2008). One useful exception is white OLEDs, since exciplex featured in color-adjustable and very broad PL/EL spectra (Chao and Chen, 1998). However, restricted by its low PLQE, the corresponding device performance was very limited.

Exciplex was confirmed to be intermolecular TADF materials due to its extremely low HOMO-LUMO overlap and thus low exchange energy ΔE_ST_ (0–100 meV) (Graves et al., 2014). Figure 1C depicted PL/EL transient decay results of a typical exciplex couple of 4,4′,4^′′^-tris[3-methylphenyl(phenyl)amino] triphenylamine (m-MTDATA) and 2-(biphenyl-4-yl)-5-(4-tert-butylphenyl)-1,3,4-oxadiazole (t-Bu-PBD), which was characteristic of the distinct delay fluorescent component (Goushi et al., 2012). Their difference, was due to high initial triplet excitons formed on exciplex emitters under EL driving, e.g., triplets/(singlets+triplets) ~0.75 in general, which enhanced the delayed fluorescence component significantly. As indicated, its RISC rate and efficiency (*k*_RISC_, *Φ*_RISC_) were very efficient. Along with the explorations of novel exciplex emitters with enhanced PLQE, big progress has been achieved, e.g., even close to the best TADF or phosphorescent OLEDs in performance (Goushi et al., 2012; Hung et al., 2013, 2014; Liu et al., 2015a,c). Alternatively, considering the unique merits of exciplex couple, e.g., the lowest HOMO-LUMO transport gap under a certain T_1_ energy, bipolar transporting properties and triplet-to-singlet upconversion capability via RISC process, exciplex has also been used as host OLEDs to obtain ultrahigh EQE/PE performance. A representative example is a bulk exciplex-forming cohost in phosphorescent- and TADF-doped OLEDs (PhOLEDs, TADF OLEDs) by Kim et al., e.g., achieving a high EQE/PE of 29.1%/124 lm W^−1^ with slow roll-off (Park et al., 2013; Lee et al., 2014; Sun et al., 2014), and exciplex-sensitized fluorescent OLED (FOLEDs) (peak EQE/PE of 14.5%/46.1 lm W^−1^) and hybrid white OLEDs (forward-viewing peak EQE/PE of 25.5%/84.1 lm W^−1^) by Liu et al. (2015a,b). Compared to bulk exciplex, in which the constituents are physically blended and doped with guests, an interfacial exciplex host is a simplified bilayer structure doped with guests in either layer or both. Recent results show that an interfacial exciplex host can also be well-applied to OLEDs (Park et al., 2011; Seino et al., 2014; Wang et al., 2015; Zhang et al., 2016; Xu et al., 2017; Lin et al., 2018), with some unique characteristics (Al Attar and Monkman, 2016; Chen et al., 2016; He et al., 2016; Nakanotani et al., 2016; Lin et al., 2017). The key results of reported OLEDs using interfacial exciplex host are summarized in the Supplementary Materials.

The schematic energy transfer (ET) mechanisms on PhOLEDs, TADF OLEDs and FOLEDs using the interfacial exciplex as a sensitizing host are shown in Figures 1D–F (Park et al., 2011; Seino et al., 2014; Liu et al., 2015a,b; Wang et al., 2015; Zhang et al., 2016). The sensitizing function of the exciplex host is ascribed to its TADF property, in which initial populations of triplets on them are efficiently up-converted into singlets, followed by Förster ET to a guest. While the guest is phosphorescent or TADF emitters, such a process is unique in lowering local exciton density of long-lived triplets on guests, therefore sharply increasing EQE/PE performance and also distinctly alleviating triplet-involved quenching processes, e.g., triplet-triplet annihilation (TTA), triplet-polaron annihilation (TPA) etc (Moon et al., 2017). Due to the RISC process in the exciplex host, enhanced Förster ET is believed to be the main PL/EL ET mechanism. However, debates still exist (Seino et al., 2014; Zhou et al., 2014), i.e., Förster or Dexter ET or both. In some reports the doping concentration in PhOLEDs and TADF OLEDs is as high as 10–20 wt.% (Seino et al., 2014; Wang et al., 2015; Liu et al., 2016; Lin et al., 2018). Dexter ET is thus unavoidable, playing positive roles on triplet-harvesting. However, in FOLEDs (Figure 1F), such Dexter ET, also trap-assisted recombination on guest itself, should be well-removed (Nakanotani et al., 2014; Zhang et al., 2014; Liu et al., 2015b), which was realized by minimizing doping concentration of guest and novel guest (Zhang et al., 2018).

Seino et al. (2014) firstly reported blue PhOLEDs via an interfacial exciplex host, in which a combination of di-[4-(N,N-ditolyl-amino)-phenyl]cyclohexane (TAPC) and home-made 5′,5^′*′′′*^-sulfonyl-di-1,1′:3′,1″-terphenyl (BTPS) was used as the exciplex (S_1_/T_1_: 2.97/2.82 eV) and blue phosphor phosphoriridium(III) bis[(4,6-difl uorophenyl)-pyridinate- N,C^2^′]picolinate (FIrpic) (S_1_/T_1_: 2.78/2.77 eV) was doped into the BTPS layer. Direct exciton confinement occurred at the TAPC/BTPS exciplex interface, followed by Förster and Dexter ET to the dopant. It achieved a satisfied PE_100_ of 50.1 lm W ^−1^ merely at a low voltage of 2.90 V, along with an ultralow turn-on voltage (V_on_) of 2.5 V. Compared to the control device, without using the exciplex host, the efficiencies were moderately enhanced, but the turn-on/driving voltages were dramatically lowered, e.g., 2.80 → 2.50 V, 3.32 → 2.90 V for V_on_/V_100_. They further developed power-efficient TADF OLEDs with a similar structure (Seino et al., 2016). For example, the constructed EML consisted of CBP:5 wt.% 4CzIPN/B4PYMPM, in which 4,4′-N,N′-dicarbazolylbiphenyl (CBP)/B4PYMPM was an interfacial exciplex couple and 1,2,3,5-tetrakis (carbazol-9-yl)-4,6-dicyanobenzene (4CzIPN) was the TADF emitter. The charge and exciton formation route were analogous to that of PhOLEDs, and the EL mechanism is shown in Figure 1E. The resultant device displayed ultralow V_on_ of 2.33 V, EQE_max._/PE_max._ of 25.7% and 106.9 lm W^−1^, slightly declined to 24.8% and 79.4 lm W^−1^ at high luminance of 1,000 cd m^−2^. Such performance came close to the best PhOLED counterpart ever developed.

Duan's group provided an illustration on applications of the exciplex host (Zhang et al., 2016), and provided direct comparisons on the interfacial exciplex vs. the bulk exciplex host, from the viewpoint of device efficiencies, roll-off performance and device lifetime. The constituting materials were one bipolar host (3′-(4,6-diphenyl-1,3,5-triazin-2-yl)-(1,1′-biphenyl)-3-yl)-9-carbazole (CzTrz), and a donor molecule tris(4-(9H-carbazol-9-yl)phenyl)amine (TCTA) to form the CzTrz:TCTA bulk exciplex or CzTrz/TCTA interfacial exciplex host, respectively, where orange phosphor was (acetylacetonato)bis[2-(thieno[3,2-c]pyridin-4-yl)phenyl]-iridium(III) (PO-01). Very surprisingly, the interfacial exciplex host based PhOLED comprehensively outperformed the bulk excipelx host based PhOLED. EQE_max._/EQE_5000_/EQE_10000_ and PE_max._/PE_5000_/PE_10000_ of the former device reached to 27.0/25.6/24.0% and 73.1/52.1/44.6 lm W^−1^, as compared to 23.5/21.5/19.5% and 58.5/41.1/33.2 lm W^−1^ achieved for the latter. Obviously, the interfacial exciplex host rendered the PhOLED much higher EQEs/PEs along with the alleviated roll-off rate. As disclosed, it was due to the enhanced Förster ET from the CzTrz/TCTA to the dopant in the EML [CzTrz:PO-01(1–3 wt.%)]. Despite relatively high local exciton density at the exciplex interface, fast and efficient long-range Förster ET spread these excitons throughout the EML, thereby overcoming TTA, TPA quenching limitations. Besides, with the CzTrz/TCTA interfacial exciplex host, the device lifetime of PhOLED was enhanced by almost two orders of a magnitude compared to the device counterpart with a bulk exciplex host (L_0_:1,000 cd m^−2^), since this structure avoided the formation of easily dissociated high-energy aromatic amines, TCTA in this case, donor excited states (Zhang et al., 2016). The formation possibility of unstable high-energy TCTA excitons was largely lowered in the interfacial exciplex host device structure, which was indicated by a condition experiment, i.e., a much longer device lifetime using a lower content of the TCTA constitute. It was not mentioned why the bulk exciplex host based PhOLEDs exhibited much lower efficiencies (as well as quicker roll-off rates), compared to the interfacial exciplex host PhOLEDs. Probably, the TCTA-excitonic involved a degradation process. As indicated, accelerated TPA and/or TTA quenching also distinctly deteriorated device efficiencies and roll-off behaviors. In short, this report might have referential significance in solving efficiency and lifetime issues of other OLEDs with host-guest structures that were not mentioned. The same group further used such geometry in red PhOLEDs, in which the exploration of constituting materials to form suitable interfacial exciplex was proven to be important (Song et al., 2018).

Xu et al. (2017) further reported a new emitting sub-unit design in tandem OLEDs, using an ultra-thin emissive layer (UEML), i.e., green-color phosphor bis[2-(2-pyridinyl-N)phenyl-C](acetylacetonato)-iridium(III) [Ir(ppy)_2_(acac)], sandwiched between a layer of 1,1-bis[(di-4-tolylamino)phenyl]cyclohexane (TAPC) and a layer of 1,3,5-tri(p-pyrid-3-yl-phenyl)benzene (TmPyPB), in which TAPC/TmPyPB could form an interfacial exciplex. It displayed a peak LE/EQE of 135.74 cd A^−1^/36.85%, which is among the best efficiencies of OLEDs using non-doped EML without using an out-coupling method. Despite indirect contact, exciplex excited states were generated efficiently via long-range coupling under device operation (Al Attar and Monkman, 2016; Nakanotani et al., 2016), followed by sufficient Förster/Dexter ET to the UEML. As the complexed co-evaporation operation is avoided, such architecture is significant in simplifying the manufacturing process of OLEDs and enhancing yield and repeatability of OLED products.

Su's group conduced systematic works on interfacial exciplex host application in FOLEDs (Li et al., 2018). FOLEDs with different EML structures were fabricated, i.e., TAPC:1% DBP/TmPyTz (device 1), TAPC:1% DBP/TAPC(3 nm)/TmPyTz (device 2), TAPC:1% DBP/mCP(3 nm)/TmPyTz (device 3), in which TAPC/TmPyTz formed exciplex directly at the interface (device 1 and 2) or even long-range distance (device 3, with mCP spacer) (see the Supplementary Materials for a detailed performance) and DBP was a common fluorescent emitter. Among all of them, device 3 with a spatially separated exciplex couple host was the best, i.e., simultaneously achieving a low driving voltage, a high luminance and efficiency, e.g., V_on_/L_max._/EQE_max._/PE_max_ of 2.18 V, 2956.8 cd m^−2^, 14.8% and 38.8 lm W^−1^. As illustrated, the merits of such FOLEDs using a spatially separated exciplex host lie in separating exciton generation and energy transferring areas, and also restraining the charge trapping effect of the dopant emitter, which is basically different from common FOLEDs.

Moreover, Lin et al. (2017) successfully constructed prototypical up-conversion OLEDs on the basis of a so-called exciplex-sensitized TTA (ESTTA) mechanism, featuring ultra-low sub-bandgap EL driving voltages, e.g., light turn-on merely at 2.2 V for high energy blue emission (2.9 eV for its bandgap). As verified, low-energy exciplex triplets formed at the interface of 4, 4′, 4″ -tris[3-methylphenyl(phenyl)amino]triphenylamine (m-MTDATA)/9,10-bis(2′-naphthyl) anthracene (ADN) are harvested by AND themselves and then trigger their TTA processes to realize high-energy blue emission (S_1_→*S*_0_). At first glance, such ESTTA-OLED featured in low-voltage driving but suffered from low EQE performance (0.1%) due to back energy transfer quenching from S_1_ of ADN to S_1_ of the exciplex. After incorporating the “triplet diffusion and singlet blocking (TDSB)” layer and/or a more efficient fluorescent dopant, e.g., DPAVBi, the corresponding EQE performance was sharply enhanced to 3.8%. Impressively, for TTA emissive material, this configuration theoretically requires only one-half of the driving voltage equal to its singlet photonics energy. This work provides a novel clue toward developing ultralow driving-voltage and power-efficient OLEDs (especially for the blue one).

## Interfacial Exciplex as Host in Solution-Processed OLEDs

Solution-processed OLEDs (s-OLEDs) are appealing since the adopted wet-process approach is cost-effective, and more suitable for future flexible, stretchable and large-size displaying and lighting applications via high-speed printing, “roll-to-roll” coating industrial techniques. However, one of the unsolved challenges lies in how to realize sufficient high EL performance, especially low power consumption. To this goal, among various strategies, interfacial exciplex was found to be an ideal host in various types of s-OLEDs, i.e., acquiring sufficient low driving condition, high PE while using a very simplified device structure.

**Figure 2:**
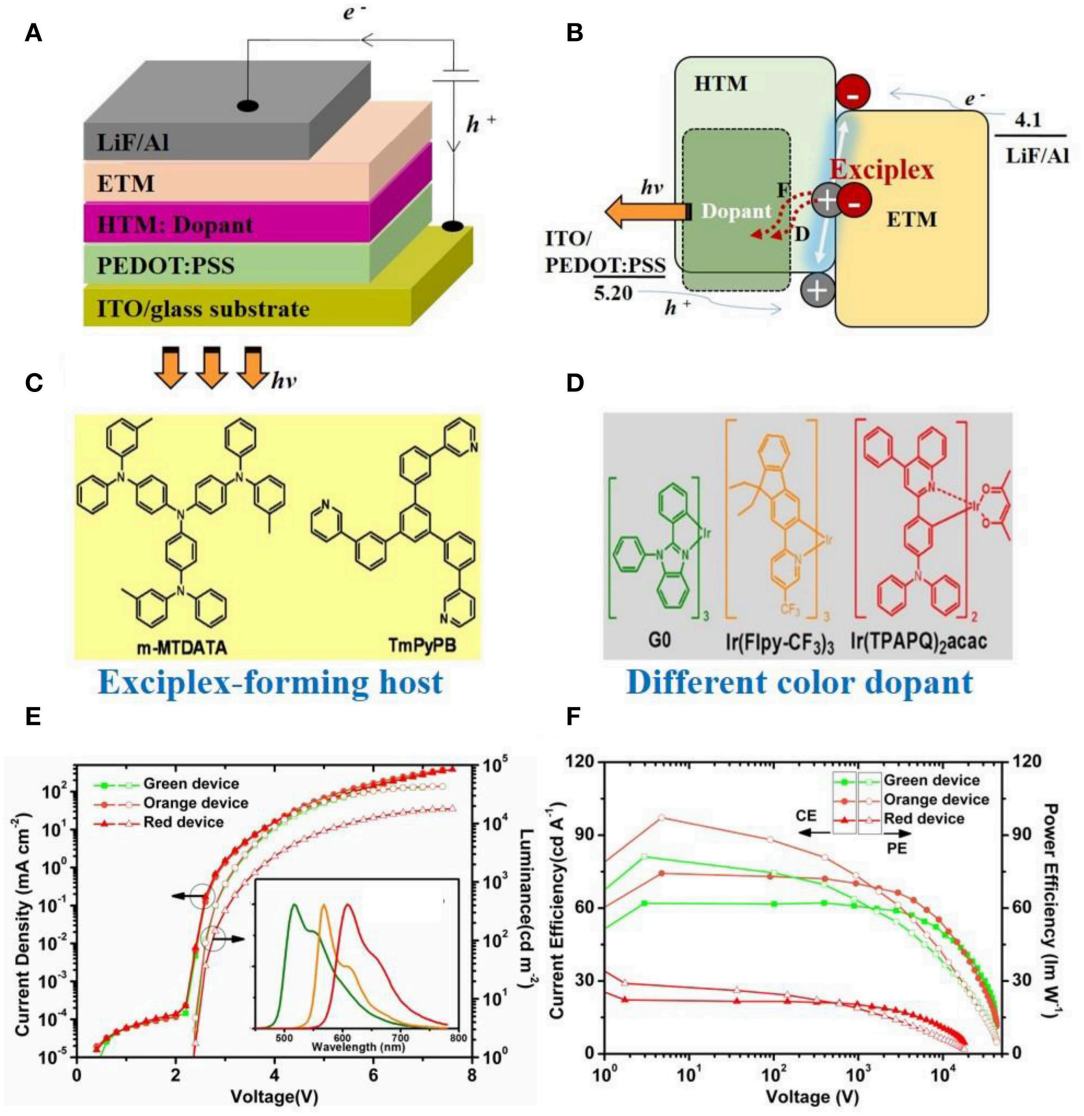
**(A)** Device structure of s-OLEDs with interfacial exciplex host. **(B)** Schematic EL driving mechanisms for them, in which F, D denotes Förster and Dexter ET, respectively shown in Wang et al. (2015) and Liu et al. (2018a). **(C)** chemical structures of the m-MTDATA:TmPyPB exciplex couple. **(D)** Example of dopants used with different colors. **(E,F)** J-V-L and LE-V-PE characteristics of G/O/R color s-PhOLEDs illustrated in Wang et al. (2015).

Zhang and Wang et al. first reported power-efficient phosphorescent s-OLEDs (s-PhOLEDs) using an interfacial exciplex host, i.e., m-MTDATA/TmPyPB (Wang et al., 2015). The achieved PE_max._ of orange s-PhOLED reached a record value of 97.2 lm W^−1^, along with ultra-low V_on_ of 2.36 V, and extremely low driving voltages of 2.60/3.03 V at the luminance of 100/1,000 cd m^−2^. Such PE is the best among all-reported s-OLEDs and even superior to thermal-evaporated PhOLEDs with the same color. Figures 2A,B depicts the device structure and the proposed EL driving mechanisms, in which both holes and electrons were barrier-freely injected, transported and then combined at the m-MTDATA/TmPyPB heterojunction to form exciplex excitons, and then transferred to dopants of the EML via Förster/Dexter ET. A negligible influence on current density-voltage (J-V) characteristics was observed by doping the guests, indicating that notorious charge trapping/scattering effects of dopants (Wang et al., 2018) were absent in such an architecture. By contrast, s-PhOLEDs using bulk exciplex of m-MTDATA:TmPyPB (1:1 w/w) was unsatisfied, corresponding to a low PE_max._ of 35.2 lm W^−1^ mainly due to sharply increased driving voltages, e.g., V_on_/V_100_/V_1000_: 4.15/5.18/5.80 V. Two reasons were involved; i) low charge transporting capability of the bulk exciplex couple due to their intrinsic incompatibility (Yao et al., 2018); ii) the serious charge trapping tendency of dopant in the EML structure of m-MTDATA:TmPyPB:dopant (Wang et al., 2015). Accordingly, interfacial exciplex rather than bulk exciplex shown here is more suitable for s-PhOLEDs as a host. By replacing the orange phosphor to other ones (Figures 2C–F), power-efficient s-PhOLEDs with similar EL driving features were realized, e.g., 81.1 lm W^−1^ for green (Wang et al., 2015) and 44.5 lm W^−1^ for red (Liu et al., 2016, 2018a), respectively, confirming its universal application potential.

Komatsu et al. (2015) fabricated efficient TADF s-OLEDs using 4CzIPN as the dopant dispersed within the CBP matrix, and an interfacial exciplex couple of CBP (35 nm)/bis-4,6-(3,5-di-4-pyridylphenyl)-2-methylpyrimidine (B4PyMPM) as the host. With the analogous ET mechanisms shown in Figure 1E and EL driving process shown in Figure 2B, the device was also satisfied in performance, achieving a very low V_on_ of 2.5 V and high PE of 55 lm W^−1^. It was one of the best ever developed TADF s-OLEDs with a TADF small molecular emitter (Kim et al., 2016; Liu et al., 2018b). On the topic of TADF polymer s-OLEDs (Nikolaenko et al., 2015; Nobuyasu et al., 2016; Zhang and Cheng, 2019), the interfacial exciplex host strategy was also confirmed as a wise choice. For instance, the application of interfacial exciplex TAPC/TmPyPB as the host of polymer PAPTC endowed the device with a very satisfied overall EL performance, with extremely low voltages of 2.50/2.91/3.51 V at a luminance of 1/100/1,000 cd m^−2^, high peak EQE/PE of 14.9%/50.1 lm W^−1^, and a wonderful slow roll-off rate, of J_50_ of 63.16 cm^−2^ and L_50_ of approx. fifteen thousand cd m^−2^ (Lin et al., 2018). It is distinctly superior to that of a control device using pure PAPTC EML. Especially, efficiency roll-off was enhanced nearly 3-fold. Further studied disclosed that, with respect to the pure PAPTC layer, the optimized structure of TAPC:PAPTC (20 wt.%)/TmPyPB not only gained a much higher PLQE (79.5 vs. 36.3%) by largely restraining aggregation-induced Dexter triplet-quenching (Lee et al., 2017), but also sharply reduced triplet population on PAPTC itself by 4-fold enhancement of its *k*_RSIC_ to as high as 1.48 × 10^7^ s^−1^ (Moon et al., 2017). These two aspects were combined to explain the promotions of the EL performance presented.

## Concluding Remarks

Over the past several years, successful applications of an interfacial exciplex host in OLEDs were presented. Due to its merits in barrier-free exciton generation, “Well”-like exciton confinement and high ET efficiencies, simultaneous low voltage, high EQE/PE, low roll-off rate and even much higher device stability were representatively achieved, irrespective of dopant types. It should be strengthened that, compared to a traditional host, RISC up-conversion of the exciplex host renders the interfacial exciplex with an enhanced Förster ET process, guaranteeing low exciton density on emitters, thereby removing the risk of accelerating exciton-aggregation quenching. Moreover, local high charge/exciton density at the interfacial exciplex region is not a drawback but a special advantage that is not accessible in the bulk exciplex counterpart. For instance, a simple manipulation of the category of the major carrier (hole-rich or electron-rich or balanced) at the donor/accepter heterojunction, was found to induce exciplex recombination or Auger recombination or both (He et al., 2016). After doping with an appropriate guest, the novel EL driving mechanism is anticipated and may be significant. Unfortunately, the corresponding phenomena and mechanisms were not found in bulk exciplex counterparts. In addition, an interesting long-ranging coupling property of exciplex has been discovered recently but has still not been widely used in host applications (Al Attar and Monkman, 2016; Nakanotani et al., 2016). In this respect, interfacial exciplex architecture is also an ideal choice.

It must be noted that compared to bulk exciplex, relevant interfacial exciplex host applications in OLEDs are still limited and required. It is believed that if the structural advantages of interfacial exciplex are further utilized, there are more opportunities to construct OLEDs with a much higher performance and can also enrich our understanding of the related physical processes. Both of them are crucial from an OLED science and technology point of view.

## Author Contributions

All authors listed have made a substantial, direct and intellectual contribution to the work, and approved it for publication.

### Conflict of Interest Statement

The authors declare that the research was conducted in the absence of any commercial or financial relationships that could be construed as a potential conflict of interest.
