# Supplementary material for: Recent Applications of Interfacial Exciplex as Ideal Host of Power-Efficient OLEDs
Source: Front Chem. 2019 May 7;7:306. doi: 10.3389/fchem.2019.00306 (PMC6514091; doi:10.3389/fchem.2019.00306)
Supplement: Supplementary file 1 [file Data_Sheet_1.pdf]

Supplementary Material

Supplementary Table 1. Summary of device performance parameters for state-of-the-art OLEDs using interfacial exciplex as host, including both thermal-evaporated OLEDs (e-OLEDs) and solution-processed OLEDs (s-OLEDs), respectively.

Type	EML	$V_{on/1000}$ [V]	$L_{max.}$ [cd/m^2]	$EQE_{max./1000}$ [%]	$PE_{max./1000}$ [lm/W]	CIE [x,y]	Ref.
**e-OLED**, in which e-**FOLED, e-PhOLED** and **e-TADF OLED** were classified by their used dopant, i.e. fluorescent, phosphorescent or TADF emitter, respectively.							
e-FOLED	TAPC:1%DBP/TmPyTz	2.18/-	520.3	10.1/-	17.8/-	0.62,0.38 [a]	[1]
	TAPC:1%DBP/TAPC(3nm) /TmPyTz [b]	2.18/-	457.1	14.5/-	42.9/-	0.53,0.46 [a]	[1]
	TAPC:1%DBP/mCP(3nm)/ TmPyTz [c]	2.18/-	2956.8	14.8/-	38.8/-	0.54,0.45 [a]	[1]
	TCTA/rubrene(0.2nm)/TP Bi	3.1/-	18310	-/-	12.7/-	[d]	[2]
e-PhOLED	CBP:6 mol.% Ir(ppy)$_3$/B3PYMPM	-/-	-	20.1/-	-/-	[e]	[3]
	TAPC/BTPC:11 wt.% FIrpic	2.5/3.4	-	-/17.2	-/33.8	[f]	[4]
	TCTA/CzTrz: 3% PO-01[i]	-/-	-	27.0/25.6[g]	73.1/52.1[g]	[h]	[5]
	TAPC:o-DTPPC: 6 wt.% Ir(mphmq)$_2$tmd	4.35	>15000	21.0/18.8[g]	18.0/12.6[g]	[j]	[6]
	TAPC/Ir(ppy)$_2$(acac) (0.8nm)/TmPyPB [k]	5.76[l]/-	~ 40000	36.9/-	60.0/-	0.35,0.61	[7]

	TCTA/$(tbt)_2$Ir(acac) (0.3 nm) /B3PYMPM	3.2/-	17400	19.5/12.0	53.1/-	[m]	[8]
e-TADF OLED	CBP: 5 wt.% 4CzIPN/B4PyPPM	2.33/	-	25.7/24.8	106.9/79.4	[n]	[9]
	CBP: 20 wt.% DACT-II/B4PyMPM	2.30	>10000	27.6/21.7	124.5/76.5	[o]	[10]
**s-OLED**, in which s-**FOLED, s-PhOLED** and **s-TADF OLED** were classified by their used dopant, i.e. fluorescent, phosphorescent or TADF emitter, respectively.							
s-FOLED	-	-	-	-	-	-	-
s-PhOLED	m-MTDATA: 1 wt.% Ir(Flpy-CF_3)$_3$/TmPyPB	2.36/3.03	43085	25.2/23.7	97.2/72.5	0.52,0.47	[11]
	m-MTDATA: 10 wt.% G0/TmPyPB	2.36/3.03	41539	18.1/17.8	81.1/62.5	0.35,0.59	[11]
	m-MTDATA: 5 wt.% Ir(TPAPQ)$_2$(acac)/TmPyPB	2.36/3.42	17902	16.3/14.9	29.0/18.8	0.64,0.36	[11]
	m-MTDATA: 3 wt.% Ir(DPA-Flpy-CF_3)$_2$acac/TmPyPB	2.3/2.8 [p]	~ 10000	19.3/19.0[p]	44.5/36.2[p]	0.64,0.36	[12]
	m-MTDATA: 3 wt.% Ir(Flpy-CF_3)$_2$acac/TmPyPB	2.2/2.6 [p]	23005	23.7/-	80.4/-	0.56,0.43	[13]
s-TADF OLED	CBP: 5 wt.% 4CzIPN/B4PyMPM	2.5/4.7	-	16[p]/13	55/27	[n]	[14]
	TAPC:20 wt.% PAPTC/TmPyPB	2.50/-	26321	14.9/14.3	50.1/-	0.34,0.56	[15]
	DCzDCN:SimCP2(2:1):10 wt.% TXO-TPA/CDBP(3.5 nm)/B4PYPPM	-	-	10.02/-	20.71/-	0.32,0.34 [q]	[16]

Note:

[a]: at 1mA cm^{-2}. Because of incomplete energy transfer from TAPC/TmPzTz interfacial exciplex host to DBP, EL spectra of e-OLEDs were varied as increasing the driving voltage(or current density or luminance);

[b]: exciton formation region was dominantly formed at the TAPC/TmPyTz interface, followed by Förster energy transfer to DBP emitter throughout pure TAPC(3nm) layer;

[c]: actual exciplex host was long-range charge-transfer couple of TAPC and TmPyTz, in which mCP functioned as spacer;

[d]: EL spectra of OLEDs displayed the whole emission from rubrene emitter, in which λ_{EL} located at 561 nm;
[e]: purely from the green $Ir(ppy)_3$ emitter;
[f]: purely from the blue FIrpic emitter;
[g]: at the luminance of 5000 cd/m^2;
[h]: purely from the orange PO-01 emitter;
[i]: in this work, device lifetime, efficiency and roll-off behaviors of PhOLEDs using interfacial or bulk exciplex host were systematically compared in parallel, confirming the superiorities of interfacial exciplex host in optimizing all of these critical device parameters;
[j]: purely from the red $Ir(mphmq)_2tmd$ emitter;
[k]: interfacial exciplex couple was TAPC/TmPyPB, and the device was two-unit tandem PhOLED;
[l]: at 0.2 mA/cm^2;
[m]: purely from the orange $(tbt)_2Ir(acac)$ emitter;
[n]: purely from the green 4CzIPN TADF emitter;
[o]: purely from the green DACT-Ⅱ TADF emitter;
[p]: at a luminance of 100 cd/m^2.
[q]: at a luminance of 500 cd/m^2. The EL spectra consisted of blue-orange complementary two colors for white emission, in which blue and orange component came from interfacial exciplex of CDBP/B4PYPPM (λ_{EL} : 445nm and TXO-TPA emitter (via partial Förster Energy transfer from the interfacial exciplex, i.e. λ_{EL} : 568 nm), respectively.

Reference:

[1] B. B. Li, L. Gan, X. Y. Cai, X. L. Li, Z. H. Wang, K. Gao, D. C. Chen, Y. Cao, S. J. Su, *Adv. Mater. Interfaces* **2018**, *5*, 1800025.

[2] X. Wang, R. Wang, D. L. Zhou, J. S. Yu, *Synth. Met.* **2016**, *214*, 50-55.

[3] Y.-S. Park, W.-I. Jeong, J.-J. Kim, *J. Appl. Phys.* **2011**, *110*, 124519.

[4] Y. Seino, H. Sasabe, Y.-J. Pu, J. Kido, *Adv. Mater.* **2014**, *26*, 1612-1616.

[5] D. Zhang, M. Cai, Y. Zhang, Z. Bin, D. Zhang, L. Duan, *ACS Appl. Mater. Interfaces* **2016**, *8*, 3825-3832.

[6] X. Song, D. Zhang, T. Huang, M. Cai, L. Duan, *Sci. China-Chem.* **2018**, *61*, 836-843.

[7] T. Xu, J.-G. Zhou, C.-C. Huang, L. Zhang, M. K. Fung, I. Murtaza, H. Meng, L.-S. Liao, *ACS Appl. Mater. Interfaces* **2017**, *9*, 10955-10962.

[8] Y. G. Qi, S. H. Hou, J. Li, H. Guo, J. S. Yu, *J. Lumin.* **2017**, *192*, 1242-1249.

[9] Y. Seino, S. Inomata, H. Sasabe, Y. J. Pu, J. Kido, *Adv. Mater.* **2016**, *28*, 2638.

[10] H. Sasabe, R. Sato, K. Suzuki, Y. Watanabe, C. Adachi, H. Kaji, J. Kido, *Adv. Opt. Mater.* **2018**, *6*, 1800376

[11] S. Wang, X. Wang, B. Yao, B. Zhang, J. Ding, Z. Xie, L. Wang, *Sci. Rep.* **2015**, *5*, 12487.

[12] X. Liu, B. Yao, Z. Zhang, X. Zhao, B. Zhang, W.-Y. Wong, Y. Cheng, Z. Xie, *J. Mater. Chem. C* **2016**, *4*, 5787.

[13] X. Liu, B. Yao, H. Wang, B. Zhang, X. Lin, X. Zhao, Y. Cheng, Z. Xie, W.-Y. Wong, *Org. Electron.* **2018**, *54*, 197-203.

[14] R. Komatsu, H. Sasabe, S. Inomata, Y.-J. Pu, J. Kido, *Synth. Met.* **2015**, *202*, 165-168.

[15] X. Lin, Y. Zhu, B. Zhang, X. Zhao, B. Yao, Y. Cheng, Z. Li, Y. Qu, Z. Xie, *ACS Appl. Mater. Interfaces* **2018**, *10*, 47-52.

[16] Y. W. Liu, X. F. Wei, Z. Y. Li, J. J. Liu, R. F. Wang, X. X. Hu, P. F. Wang, T. Qi, Y. Wang, *Adv. Opt. Mater.* **2018**, *6*, 1800978.
